# Assessment of limiting dietary amino acids in broiler chickens offered reduced crude protein diets

**DOI:** 10.1016/j.aninu.2021.11.010

**Published:** 2022-02-24

**Authors:** Craig W. Maynard, Michael T. Kidd, Peter V. Chrystal, Leon R. McQuade, Bernie V. McInerney, Peter H. Selle, Sonia Y. Liu

**Affiliations:** a Center of Excellence for Poultry Science, University of Arkansas, Fayetteville, AR, 72701, United States; b Poultry Research Foundation, Faculty of Science, The University of Sydney, 425 Werombi Road, Camden, NSW, 2570, Australia; c School of Life and Environmental Sciences, Faculty of Science, The University of Sydney, Sydney, NSW, 2006, Australia; d Australian Proteome Analysis Facility, Macquarie University, Sydney, NSW, 2109, Australia; e Sydney School of Veterinary Science, Faculty of Science, The University of Sydney, Sydney, NSW, 2006, Australia

**Keywords:** Amino acid, Broiler chicken, Crude protein, Low protein diet

## Abstract

As lowering crude protein (CP) in poultry diets continues to minimize amino acid excess, it is important to understand the limiting order of amino acids and the impact of their deficiencies. Therefore, a pair of experiments were conducted to observe the effects of individual amino acid deletions on growth performance, carcass traits, and nutrient utilization. Both experiments involved 3 control diets based on wheat and soybean meal, including a 210.0 g/kg CP industry control (IC), 186.7 g/kg CP positive control (PC) supplemented with feed-grade amino acids to match the IC amino acid profile, 186.7 g/kg CP negative control (NC) with reducing N corrected apparent metabolizable energy (AME_N_) by 0.5 MJ/kg and removing feed-grade amino acids beyond L-Lys-HCl, DL-Met, and L-Thr from PC. Ten deletion diets where the following supplemented amino acids were individually removed from the PC: Val, Ile, Leu, Trp, Arg, His, Phe + Tyr, glycine equivalence (Gly_equi_), Pro, and Energy (0.5 MJ/kg reduction in AME_N_ of the PC). All diets were formulated to contain similar concentrations of digestible Lys, total sulfur amino acid (TSAA) and Thr. Experimental diets were offered to broiler chickens from 15 to 22 d post–hatch in a cage study (Exp. 1) to gain digestibility and nutrient utilization data; whereas they were offered from 15 to 35 d post–hatch in a floor-pen study (Exp. 2) to gain performance and carcass yield data. The removal of supplemented Val, Arg, and Ile resulted in reduction on broiler performance (*P* < 0.05), and the removal of Val, Arg, Ile, and Gly_equi_ negatively influenced carcass traits (*P* < 0.05). Results from both experiments indicate that Val and Arg are co-limiting in wheat-soybean meal diets, but that Ile and Gly_equi_ may potentially limit breast and thigh development.

## 1 Introduction

As feed grade amino acids are commercialized at prices that are economically feasible for including in industry poultry diets, nutritionists are able to better supply diets containing amino acid profiles more closely representing ideal amino acid profiles (Baker, 1997). Refinement of dietary amino acid profiles allows for reductions in crude protein (CP), inclusion levels of protein-rich ingredients in the diet and nitrogen excretion in broiler chickens (Kidd et al., 2002; Waldroup et al., 2005b). As dietary CP levels are reduced, a progression of amino acids become limiting for growth and must be fortified to ideal amino acid levels to allow for optimal growth. Previous studies have implemented drastic reductions in dietary CP or specialized diets to determine the limiting order of amino acids in corn and soybean-based diets or diets created to focus on one ingredient (Edmonds et al., 1985; Fernandez et al., 1994; Wang et al., 1997; Wang and Parsons, 1998; Peter et al., 2000). These studies have displayed that the order of limiting amino acids differs by feed ingredients and total dietary composition (Kidd and Hackenhaar, 2006).

Practical reduction in CP involves 2 to 3 percentage point reductions from breeder recommendation (Chrystal et al., 2019) and understanding how these reductions impact performance and carcass traits due to the potential development of amino acid limitation is of paramount importance to ensure balanced amino acid supplementation. Not only should limitations caused by traditional essential amino acids be evaluated but also conditionally essential amino acids, such as Gly, or amino acids needed to be considered in tandem, such as Phe + Tyr instead of Phe alone. In the case of the aforementioned Phe + Tyr and glycine equivalence (Gly_equi_), it is nutritionally disingenuous to only consider the 'essential' portion of their requirements and disregard their 'non-essential' counterparts due to the sparing action observed by dietary inclusion of the non-essential portion (Almquist, 1947). As least-cost formulation software provides insight on the limiting amino acids in poultry diets, experimental determination allows for observation of potential negative effects of amino acid deficiencies on economic outcomes based on growth performance and carcass yield (Si et al., 2004).

The order of limiting amino acids of a given diet can be determined in growth studies using either an addition or deletion assay. Addition assay adds supplemented amino acids one by one into a basal diet containing minimal amount of synthetic amino acids, the cumulative response of the additions is often evaluated; whereas a fully supplemented diet is used in a deletion assay and one supplemented amino acid is taken out at one time. Of the two, deletion assays have been found to be more effective (Baker, 1989; Wang, 1997).

Whether amino acids are derived from intact protein or supplementation in feed could alter energy requirement in poultry. It was reported in human, energy requirement may vary when offering diets rich in intact protein, hydrolysed protein or non-bound amino acids (Rose et al., 1954). Later studies in poultry and rats found that decreasing energy levels resulted in decreased nitrogen retention and increased total feed intake (Munro and Naismith, 1953; Fisher and Shapiro, 1961). This may be due to animals were responding to the first limiting nutrient, increasing total feed intake in order to maintain protein intake target (Gous et al., 2018). Recent studies utilizing conventional diets have shown that a moderate increase of energy ranging from 0.29 to 0.46 MJ/kg had no effect (Maynard et al., 2019) or minor influence on feed intake and feed conversion (Gopinger et al., 2017; Johnson et al., 2020). However, increased relative fat pad weight to body weight was observed in broiler chickens offered low-CP diets (Chrystal et al., 2020a; Greenhalgh et al., 2020) which suggests surplus dietary energy may have been provided in diets containing lower CP and higher non-bound amino acids. The shift from intact protein to non-bound amino acids in low-CP diets may reduce energy requirement in broiler chickens because non-bound amino acids do not require digestion and are immediately available for absorption. Therefore, reducing dietary energy may improve carcass quality without compromising growth performance.

Various studies have determined the order of limiting amino acids in maize-soybean meal diets due to it being the most typical broiler diet worldwide (Schwartz and Bray, 1975; Edmonds et al., 1985; Holsheimer and Janssen, 1991; Fernandez et al., 1994; Maynard et al., 2020). Differences in global climates dictate the composition of broiler diets based on the availability of feedstuffs locally. Broiler diets are primarily based on wheat in Australia and Europe due to the high importation cost of maize and relative shortage of rainfall. Because of this, experimental determination of the order of limiting amino acids in wheat-based diets is of particular interest to these regions. More importantly, very few studies in the literature reported nutrient digestibility and utilization data to understand the response observed in growth performance. Therefore, 2 concurrent experiments were conducted to determine the limiting order of amino acids in a low-CP wheat-based diet, and the effects of serial amino acid deletions on energy utilization, protein digestibility, and plasma amino acid levels.

## 2 Materials and methods

### 2.1 Animal ethics

This study was approved by the Research Integrity and Ethics Administration of The University of Sydney (2019/1651).

### 2.2 Experimental diets

An industry control (IC) diet was formulated at 210.0 g/kg CP to reflect diets currently used in the Australian chicken meat industry; containing wheat, whole canola seed, and soybean meal (Table 1). The CP of this diet was then reduced by 30 g/kg but Lys, total sulphur amino acids (TSAA), and Thr levels were maintained. Consequently, non-bound amino acids were supplemented to match the amino acid profile of the IC diet for Val, Ile, Leu, Trp, Arg, His, Phe + Tyr, Gly_equi_, and Pro, as described in Waldroup et al. (2005b). These additions led to the final dietary CP of 186.7 g/kg in the positive control (PC) diet. A negative control (NC) diet was created by reducing N corrected apparent metabolizable energy (AME_N_) by 0.5 MJ/kg and removing all of the supplemental amino acids from the PC and replacing them with nonessential N in the form of Glu at the expense of filler to maintain a CP of 186.7 g/kg. Each of the supplemented amino acids or groups of amino acids (e.g., Phe + Tyr and Gly_equi_) were then serial deleted from the PC resulting in the 9 deletion diets that are identified by their respective 3-letter codes: Val, Ile, Leu, Trp, Arg, His, Phe + Tyr, Gly_equi_, and Pro. An additional diet, coded ‘Energy’, was created by reducing the energy content of the PC by 0.5 MJ/kg. Any variance in dietary volume among the treatments was brought up to 1,000 g/kg with sand. An inert marker, acid insoluble ash (AIA), was added at 20 g/kg to allow for determination of N digestibility values. All diets were mixed in a vertical screw mixer and steam pelleted at 80 °C. The extent to which the amino acid levels were reduced can be found in Table 2.

**Table 1:** Composition of experimental diets fed during the periods in both experiments containing various commercial amino acid supplementation^1^.

Item	IC	PC	NC	Val	Ile	Leu	Trp	Arg	His	Phe + Tyr	Gly_equi_	Pro	Energy
Ingredients, g/kg as-fed													
Wheat	584.6	641.7	641.7	641.7	641.7	641.7	641.7	641.7	641.7	641.7	641.7	641.7	641.7
Canola seed	40.0	40.0	40.0	40.0	40.0	40.0	40.0	40.0	40.0	40.0	40.0	40.0	40.0
Soybean meal	286.6	164.5	164.5	164.5	164.5	164.5	164.5	164.5	164.5	164.5	164.5	164.5	164.5
Soy oil	34.7	37.4	24.8	37.4	37.4	37.4	37.4	37.4	37.4	37.4	37.4	37.4	24.8
L-LysHCl	2.7	6.4	6.4	6.4	6.4	6.4	6.4	6.4	6.4	6.4	6.4	6.4	6.4
DL-Met	2.9	4.0	4.0	4.0	4.0	4.0	4.0	4.0	4.0	4.0	4.0	4.0	4.0
L-Thr	1.3	3.0	3.0	3.0	3.0	3.0	3.0	3.0	3.0	3.0	3.0	3.0	3.0
L-Val	0.0	2.2	0.0	0.0	2.2	2.2	2.2	2.2	2.2	2.2	2.2	2.2	2.2
L-Arg	0.0	3.6	0.0	3.6	3.6	3.6	3.6	0.0	3.6	3.6	3.6	3.6	3.6
L-Ile	0.0	2.1	0.0	2.1	0.0	2.1	2.1	2.1	2.1	2.1	2.1	2.1	2.1
L-Leu	0.0	3.5	0.0	3.5	3.5	0.0	3.5	3.5	3.5	3.5	3.5	3.5	3.5
L-His	0.0	1.2	0.0	1.2	1.2	1.2	1.2	1.2	0.0	1.2	1.2	1.2	1.2
Gly	0.0	1.8	0.0	1.8	1.8	1.8	1.8	1.8	1.8	1.8	0.0	1.8	1.8
L-Ser	0.0	2.2	0.0	2.2	2.2	2.2	2.2	2.2	2.2	2.2	0.0	2.2	2.2
L-Trp	0.0	0.6	0.0	0.6	0.6	0.6	0.0	0.6	0.6	0.6	0.6	0.6	0.6
L-Phe	0.0	2.3	0.0	2.3	2.3	2.3	2.3	2.3	2.3	0.0	2.3	2.3	2.3
L-Tyr	0.0	1.7	0.0	1.7	1.7	1.7	1.7	1.7	1.7	0.0	1.7	1.7	1.7
L-Pro	0.0	2.0	0.0	2.0	2.0	2.0	2.0	2.0	2.0	2.0	2.0	0.0	2.0
L-Glu	0.0	0.0	38.4	2.8	2.4	3.9	0.9	12.2	3.5	3.5	6.7	2.6	0.0
NaCl	2.5	0.0	0.0	0.0	0.0	0.0	0.0	0.0	0.0	0.0	0.0	0.0	0.0
NaHCO_3_	0.8	4.4	4.4	4.4	4.4	4.4	4.4	4.4	4.4	4.4	4.4	4.4	4.4
KHCO_3_	0.0	2.0	2.0	2.0	2.0	2.0	2.0	2.0	2.0	2.0	2.0	2.0	2.0
Limestone	8.9	8.7	8.7	8.7	8.7	8.7	8.7	8.7	8.7	8.7	8.7	8.7	8.7
CaHPO_4_	10.9	12.6	12.6	12.6	12.6	12.6	12.6	12.6	12.6	12.6	12.6	12.6	12.6
Enzyme blend^2^	0.2	0.2	0.2	0.2	0.2	0.2	0.2	0.2	0.2	0.2	0.2	0.2	0.2
Choline chloride	0.9	0.9	0.9	0.9	0.9	0.9	0.9	0.9	0.9	0.9	0.9	0.9	0.9
Celite^3^	20.0	20.0	20.0	20.0	20.0	20.0	20.0	20.0	20.0	20.0	20.0	20.0	20.0
Inert filler^4^	1.0	28.6	26.1	28.0	28.3	28.1	28.3	20.0	26.3	29.2	25.9	28.0	41.2
Premix^5^	2.0	2.0	2.0	2.0	2.0	2.0	2.0	2.0	2.0	2.0	2.0	2.0	2.0
Calculated composition, g/kg unless noted otherwise													
AME_N_, MJ/kg	12.90	12.90	12.40	12.90	12.90	12.90	12.90	12.90	12.90	12.90	12.90	12.90	12.40
CP	210.0	186.7	186.7	186.7	186.7	186.7	186.7	186.7	186.7	186.7	186.7	186.7	186.7
Ca	8.3	8.3	8.3	8.3	8.3	8.3	8.3	8.3	8.3	8.3	8.3	8.3	8.3
Available P	4.1	4.1	4.1	4.1	4.1	4.1	4.1	4.1	4.1	4.1	4.1	4.1	4.1
Na	1.8	1.8	1.8	1.8	1.8	1.8	1.8	1.8	1.8	1.8	1.8	1.8	1.8
Cl	2.7	1.8	1.8	1.8	1.8	1.8	1.8	1.8	1.8	1.8	1.8	1.8	1.8
K	9.3	8.0	8.0	8.0	8.0	8.0	8.0	8.0	8.0	8.0	8.0	8.0	8.0
DEB, mEq/kg	240	230	230	230	230	230	230	230	230	230	230	230	230
Digestible Lys	11.5	11.5	11.5	11.5	11.5	11.5	11.5	11.5	11.5	11.5	11.5	11.5	11.5
Digestible TSAA	8.7	8.7	8.7	8.7	8.7	8.7	8.7	8.7	8.7	8.7	8.7	8.7	8.7
Digestible Met	5.6	6.1	6.1	6.1	6.1	6.1	6.1	6.1	6.1	6.1	6.1	6.1	6.1
Digestible Cys	3.1	2.6	2.6	2.6	2.6	2.6	2.6	2.6	2.6	2.6	2.6	2.6	2.6
Digestible Thr	7.7	7.7	7.7	7.7	7.7	7.7	7.7	7.7	7.7	7.7	7.7	7.7	7.7
Digestible Val	8.7	8.7	6.5	6.5	8.7	8.7	8.7	8.7	8.7	8.7	8.7	8.7	8.7
Digestible Ile	8.0	8.0	5.9	8.0	5.9	8.0	8.0	8.0	8.0	8.0	8.0	8.0	8.0
Digestible Leu	13.9	13.9	10.4	13.9	13.9	10.4	13.9	13.9	13.9	13.9	13.9	13.9	13.9
Digestible Arg	12.3	12.3	8.7	12.3	12.3	12.3	12.3	8.7	12.3	12.3	12.3	12.3	12.3
Digestible Trp	2.5	2.5	1.9	2.5	2.5	2.5	1.9	2.5	2.5	2.5	2.5	2.5	2.5
Digestible Phe	9.2	9.2	6.9	9.2	9.2	9.2	9.2	9.2	9.2	6.9	9.2	9.2	9.2
Digestible Tyr	6.5	6.5	4.8	6.5	6.5	6.5	6.5	6.5	6.5	4.8	6.5	6.5	6.5
Digestible Phe + Tyr	15.8	15.8	11.7	15.8	15.8	15.8	15.8	15.8	15.8	11.7	15.8	15.8	15.8
Digestible Gly	7.5	7.5	5.7	7.5	7.5	7.5	7.5	7.5	7.5	7.5	5.7	7.5	7.5
Digestible Ser	9.0	9.0	6.8	9.0	9.0	9.0	9.0	9.0	9.0	9.0	6.8	9.0	9.0
Digestible Gly_equi_	13.9	13.9	10.5	13.9	13.9	13.9	13.9	13.9	13.9	13.9	10.5	13.9	13.9
Digestible His	4.8	4.8	3.6	4.8	4.8	4.8	4.8	4.8	3.6	4.8	4.8	4.8	4.8
Digestible Pro	13.3	13.3	11.3	13.3	13.3	13.3	13.3	13.3	13.3	13.3	13.3	11.3	13.3
Analyzed composition, g/kg													
CP	230.2	204.3	218.3	206.5	214.5	212.5	199.0	207.5	228.0	206.6	210.2	213.5	232.8
Total Lys	12.7	11.1	13.6	12.5	12.2	13.3	11.2	12.4	11.2	12.2	11.8	13.5	13.5
Total Met	4.1	4.4	5.3	5.1	4.9	5.2	4.7	5.0	4.8	4.8	4.7	5.4	4.8
Total Thr	9.0	8.0	8.9	8.7	8.5	8.9	8.0	8.5	8.1	8.4	8.2	8.7	9.2
Total Val	10.4	9.7	8.3	8.1	10.1	10.4	9.6	10.0	9.7	9.9	10.0	10.4	11.1
Total Ile	9.4	8.5	7.4	8.8	7.1	9.0	8.4	8.8	8.5	8.7	8.6	8.9	9.7
Total Leu	16.3	15.3	12.9	15.6	15.6	12.8	15.1	15.6	15.2	15.5	15.4	15.7	17.2
Total Arg	12.8	11.6	9.9	12.4	12.3	13.1	11.7	9.3	11.9	12.2	12.1	13.0	14.0
Total Phe + Tyr	15.2	14.3	12.2	14.8	14.7	15.4	14.5	14.9	14.6	11.6	14.7	15.0	16.5
Total Phe	10.7	10.1	8.5	10.3	10.3	10.6	10.0	10.4	10.1	8.2	10.2	10.3	11.3
Total Tyr	4.5	4.2	3.6	4.5	4.4	4.8	4.5	4.5	4.5	3.4	4.5	4.7	5.2
Total Gly	9.1	8.1	7.2	8.7	8.7	8.9	8.1	8.6	8.2	8.5	6.9	9.2	9.5
Total Ser	10.8	9.7	8.7	10.1	10.2	10.4	9.6	10.1	9.8	10.0	8.3	10.3	11.0
Total His	5.6	5.2	4.5	5.3	5.3	5.5	5.2	5.4	4.3	5.3	5.3	5.4	5.9
Total Pro	15.1	14.8	13.1	15.0	15.0	14.9	14.7	14.9	15.0	14.7	14.8	12.7	15.2
Total Glu	49.1	41.7	81.3	47.1	44.6	45.5	42.0	52.9	44.5	44.1	47.0	43.4	43.7
Total Asp	19.0	12.9	13.8	13.3	12.9	13.5	12.5	12.9	12.7	12.9	13.0	13.2	16.1
Total Ala	8.6	6.4	6.6	6.4	6.3	6.5	6.2	6.3	6.3	6.3	6.4	6.4	7.4
PDI, %	82.60	84.03	93.93	83.33	84.40	83.37	87.90	85.47	81.87	79.93	81.90	82.53	91.63

Gly_equi_ = glycine equivalence; PDI = pellet durability index.

1 Dietary treatments included 3 control diets with industry control (IC), positive control (PC), and negative control (NC). Subsequent 9 diets were identified by their deleted amino acid 3-letter codes. The ‘Energy’ diet was created by reducing the energy content of the PC by 0.5 MJ/kg. Treatment means represented average of 6 replicate cages.

2 The enzyme blend contained (per kilogram of diet): phytase, 1,000 FTU; xylanase, 4,000 BXU.

3 Celite served as a source of acid insoluble ash to provide an inert internal dietary marker to determine amino acid digestibility coefficients (World Minerals Inc, Lompoc, CA).

4 Washed builder's sand.

5 The premix provided per kilogram of complete feed: retinol, 12,000 IU; cholecalciferol, 5,000 IU; tocopherol, 50 mg; menadione, 3 mg; thiamine, 3 mg; riboflavin, 9 mg; pyridoxine, 5 mg; cobalamin, 0.025 mg; niacin, 50 mg; pantothenate, 18 mg; folate, 2 mg; biotin, 0.2 mg; copper, 20 mg; iron, 40 mg; manganese, 110 mg; cobalt, 0.25 mg; iodine, 1 mg; molybdenum, 2 mg; zinc, 90 mg; selenium, 0.3 mg.

**Table 2:** Effect of CP reduction and feed-grade amino acid supplementation on the calculated amino acid concentration of the industry control (IC), positive control (PC), and negative control (NC) diets.

Item^1^	Ratio to Lys, %			Relative to PC, %			Relative to requirement^2^, %		
	IC	PC	NC	IC	PC	NC	IC	PC	NC
Lys	100	100	100	100	100	100	100	100	100
TSAA	76	76	76	100	100	100	105	105	105
Met	49	53	53	92	100	100	122	133	133
Cys	27	22	22	119	100	100	84	70	70
Thr	67	67	67	100	100	100	100	100	100
Val	76	76	57	100	100	75	98	98	73
Ile	70	70	51	100	100	74	104	104	77
Leu	121	121	90	100	100	75	111	111	83
Trp	22	22	17	100	100	76	128	128	97
Arg	107	107	76	100	100	71	102	102	72
His	42	42	31	100	100	75	119	119	89
Phe + Tyr^3^	137	137	102	100	100	75	130	130	97
Phe	80	80	60	100	100	75	133	133	100
Tyr	57	57	42	100	100	74	126	126	93
Gly_equi_^4^	121	121	92	100	100	76	54	54	41
Gly	65	65	50	100	100	76	37	37	28
Ser	78	78	59	100	100	76	113	113	86
Pro	116	116	98	100	100	85	63	63	53

1 Digestible basis.

2 Requirement estimates adapted from Wu (2014) (ratio to digestible Lys, 11.5 g/kg): Lys, 100; Met, 40; Cys, 32; Thr, 67; Val, 77; Ile, 67; Leu, 109; Trp, 17; Arg, 105; His, 35; Phe, 60; Tyr, 45; Gly, 176; Ser, 69; Pro, 184.

3 Sum of Phe and Tyr.

4 Glycine equivalent calculated according to the equation reported by Siegert et al. (2015b).

Samples of all experimental diets were collected and stored at 1 °C. Subsamples were then taken and analyzed for CP and amino acids (AOAC method 990.03; 994.12; 982.30; 988.15). A second feed sample was collected and used to determine pellet durability index (PDI) for each diet. One-hundred-gram samples were assessed in triplicate using a Holmen Pellet Tester (New Holmen NHP200 Series 2 Pellet Durability Tester, TekPro Ltd., Willow Park, North Walsham, Norfolk, UK) with a 3-mm screen.

### 2.3 Experiment 1 — digestibility study

A total of 312 off-sex Ross 308 male broiler chicks (parent line) were obtained from a commercial hatchery and transported to the Poultry Research Foundation poultry farm. They were offered a common starter (235.0 g/kg CP, 12.13 MJ/kg) from d 0 to 14. On d 15, 4 broilers were group weighed and randomly distributed across 78 metabolic battery cages (0.51 m × 0.51 m; 0.07 m^2^/bird) and each experimental diets were offered to 6 replicate cages from 15 to 22 d post–hatch. A 48-h total excreta collection period was conducted from d 19 to 21. Excreta was collected on individual steel trays for each pen in 24 h intervals. On d 22, pen weights and feed consumption were recorded, and all broilers were euthanized by an intravenous injection of sodium pentobarbitone to allow for digesta collection. Two digesta samples were collected from the distal half of the jejunum and ileum by squeezing the lumen contents of all 4 birds in the cage. Digesta samples were then frozen until further analysis. A lighting schedule of 18 h light: 6 h dark (18L:6D) was implemented for the duration of the study. Initial temperatures were set at 32 °C and gradually reduced to 24 °C.

### 2.4 Experiment 2 — growth study

A total of 936 off-sex male (parent line) Ross 308 broiler chicks were obtained from a commercial hatchery and fed a common starter (235.0 g/kg CP, 12.13 MJ/kg) from d 0 to 14. On d 15, 12 broilers were group weighed, randomly distributed across 78 floor pens (1.50 m × 1.50 m; 0.19 m^2^/bird), and started on 1 of 13 experimental diets offered to 6 replicate pens. Floor pens contained new wood bedding, a nipple drinker with 4 nipples, and a hanging tube feeder. On d 35, all broilers were group weighed and feed consumption was recorded to determine body weight (BW) gain, feed intake, and feed conversion ratio (FCR) during 15 to 35 d. After recording feed and pen weights, 2 broilers were randomly selected, and blood samples were collected from the brachial vein using heparinized syringes and stored on ice. Immediately following collection, blood samples were centrifuged, plasma decanted, and stored at −80 °C until submission for amino acid analysis. Additionally, 5 broilers per pen from 5 replicates were randomly selected and processed to determine total breast meat (*Pectoralis major + Pectoralis minor*) and thigh yield from the right thigh.

A lighting schedule of 23L:1D was implemented from d 0 to 7 and was adjusted to 18L:6D from d 8 to the conclusion of the trial. Initial temperatures were set at 32 °C and gradually reduced to 20 °C. Dead birds were recorded and their weights were expressed as percentage of final body weight to correct bird number for feed intake. The corrected feed intake was used to calculate FCR.

### 2.5 Chemical analyses and calculation

Apparent metabolizable energy (AME), N retention, AME_N_, and CP digestibility coefficients and disappearance rates were determined according to the procedures reported by Moss et al. (2018). Plasma samples were analyzed for amino acid content using the protocols reported by Selle et al. (2016). The methods are briefly introduced below.

Excreta were dried in a forced-air oven at 80 °C for 24 h and the gross energy (GE) of excreta and diets were determined using an adiabatic bomb calorimeter (Parr 1281 bomb calorimeter, Parr Instruments Co., Moline, IL, USA). The AME values (MJ/kg) of the diets were calculated on a dry matter basis from the following equation:

$${\text{AME}}_{\text{diet}}=\frac{\left(\text{Feed intake }\times {\text{ GE}}_{\text{diet}}\right)-\left(\text{Excreta output }\times {\text{ GE}}_{\text{excreta}}\right)}{\left(\text{Feed intake}\right)}.$$

N contents of diets and excreta were determined using a nitrogen determinator (Leco Corporation, St Joseph, MI) and N retentions calculated from the following equation:

$$\text{N retention }\left(\text{g:g}\right)=\frac{\left(\text{Feed intake }\times {\text{ Nutrient}}_{\text{diet}}\right)-\left(\text{Excreta output }\times {\text{ Nutrient}}_{\text{excreta}}\right)}{\left(\text{Feed intake }\times {\text{ Nutrient}}_{\text{diet}}\right)}.$$

AME_N_ values (MJ/kg DM) were calculated by correcting N retention to 0 using the factor of 36.54 kJ/g N retained in the body (Hill and Anderson, 1958).

N concentrations were determined as already stated and AIA concentrations were determined by the method of Siriwan et al. (1993). The apparent digestibility coefficients for starch and protein (N) in 4 small intestinal sites were calculated from the following equation:

$$\text{Digestibility coefficient }=\frac{{\left(\text{Nutrient}/\text{AIA}\right)}_{\text{diet}}-{\left(\text{Nutrient}/\text{AIA}\right)}_{\text{digesta}}}{{\left(\text{Nutrient}/\text{AIA}\right)}_{\text{diet}}}.\;$$

Protein (N) disappearance rates (g/d per bird) were deduced from the following equation:

$$\text{Nutrient disappearance rate}\left(\text{g/d per bird}\right)=\text{Feed intake}\left(\text{g/bird}\right)\text{ × Dietary nutrient}\left(\text{g/kg}\right)\text{ × Digestibility coefficient .}$$

Amino acid concentrations in diets were determined via 24 h liquid hydrolysis at 110 °C in 6 mol/L HCl followed by analysis of 16 amino acids using the Waters AccQTag Ultra chemistry on a Waters Acquity UPLC (Waters Corporation. Milford, Massachusetts). Concentrations of 20 proteinogenic amino acids in plasma taken from the brachial and anterior mesenteric veins were determined using precolumn derivatisation amino acid analysis with 6-aminoquinolyl-N-hydroxysuccinimidyl carbamate (AQC; Waters AccQTag Ultra; www.waters.com) followed by separation of the derivatives and quantification by reversed phase ultra-performance liquid chromatography (Cohen 2001). All amino acids were detected by UV absorbance.

### 2.6 Statistical analyses

Pen or cage was considered the experimental unit, and treatments were assigned to pens or cages in a randomized complete block design with pen or cage location serving as the blocking factor. The statistical model is described by the below equation:

$$Y_{hi}\;=\;\mu \;+\;\theta_{h}\;+\;\tau_{i}\;+\;\varepsilon_{hi}\;,\;$$

where $Y_{hi}$ is the random variable representing the response for treatment *i* observed in block *h*, *μ* is the overall mean, $\theta_{h}$ is the effect of the *h*th block, $\tau_{i}$ is the additive effect of the *i*th treatment, $\varepsilon_{hi}$ is the random error for the *i*th treatment in the *h*th block.

Each treatment was represented by 6 replicate cages (Exp. 1) or pens (Exp. 2) for live performance, nutrient utilization, and plasma amino acids, whereas treatments were represented by 5 replicate pens for carcass traits (Exp. 2). The data from both experiments were analysed by ANOVA using JMP Pro 15. Statistical significance was considered at *P* < 0.05. When appropriate, differences among treatments were separated using a repeated *t*-test.

## 3 Results

### 3.1 Live performance

There was no significant differences on performance in birds offered the IC and PC diets (*P* > 0.05) for both the 15 to 22 d and 15 to 35 d periods in Exp. 1 and 2, respectively (Table 3, Table 6). In comparison to PC, the removal of all supplemented amino acids, Val, and Arg significantly reduced (*P* < 0.05) BW gain in Exp. 1 and 2, whereas the removal of supplemented Ile reduced (*P* < 0.05) BW gain only in Exp. 2. Feed intake was significantly higher (*P* < 0.05) for birds fed diets devoid of Leu, Gly_equi_, Trp, and Energy than those offered the PC diet, whereas the removal of supplemented Val from the diet resulted in reduced (*P* < 0.05) feed intake in Exp. 1. In Exp. 2, all birds had similar (*P* > 0.05) feed intake to those fed the PC diet except birds offered diets devoid in Val had reduced (*P* < 0.05) feed intake. In Exp. 1, broiler chickens offered the NC diet had increased FCR than birds offered the PC diet. The removal of supplemented Val, Arg, Ile, His, and Trp significantly impaired (*P* < 0.05) feed conversion compared to birds fed the PC diet. In Exp. 2, the removal of supplemented Val, Arg, Ile, and Trp significantly increased (*P* < 0.05) FCR in comparison to the PC diet. Broilers fed the NC diet and diet devoid of Arg had the worst (*P* < 0.05) feed conversion efficiency.

**Table 3:** Live performance of Ross 308 males fed experimental diets from 15 to 22 d in Exp. 1.

Item	BW gain, kg	Feed intake, kg	FCR, g:g	Mortality, %
Treatment 1				
IC	0.788^abc^	0.966^cde^	1.226^a^	0.00
PC	0.771^bcd^	0.964^de^	1.253^abc^	0.00
NC	0.707^ef^	0.957^ef^	1.354^f^	0.00
Val	0.682^f^	0.924^f^	1.357^f^	0.00
Ile	0.740^de^	0.971^bcde^	1.315^e^	0.00
Leu	0.807^ab^	1.001^abc^	1.242^ab^	4.17
Trp	0.739^de^	1.005^ab^	1.361^f^	0.00
Arg	0.706^ef^	0.962^de^	1.365^f^	0.00
His	0.752^cd^	0.968^bcde^	1.288^de^	0.00
Phe + Tyr	0.806^ab^	0.993^abcde^	1.232^a^	0.00
Gly_equi_	0.796^ab^	1.008^a^	1.267^bcd^	0.00
Pro	0.782^abc^	0.995^abcd^	1.274^cd^	0.00
Energy	0.814^a^	1.005^ab^	1.237^a^	4.17
SEM	0.0160	0.0135	0.0104	1.634
*P*-value	<0.001	<0.001	<0.001	0.537

BW = body weight; FCR = feed conversion ratio (feed:gain ratio); Gly_equi_ = glycine equivalence.

^a-f^ Within a column, means without a common superscript were determined to be significantly different (*P* < 0.05) by a student's *t*-test.

1 Dietary treatments included 3 control diets with industry control (IC), positive control (PC), and negative control (NC). Subsequent 9 diets are identified by their deleted amino acid 3-letter codes. The ‘Energy’ diet was created by reducing the energy content of the PC by 0.5 MJ/kg. Treatment means represent average of 6 replicate cages.

**Table 4:** Nutrient utilization and excreta dry matter of Ross 308 males fed diets varying in amino acid and energy supplementation 15 to 22 d in Exp. 1.

Item	AME, MJ/kg	ME:GE ratio, MJ/MJ	N retention, g:g	AME_N,_ MJ/kg	Excreta dry matter, g/kg
Treatment 1					
IC	12.76^b^	0.726^bc^	0.663^de^	11.87^cd^	217.6^c^
PC	13.23^ab^	0.770^a^	0.732^ab^	12.35^ab^	258.7^cd^
NC	12.78^b^	0.772^a^	0.653^d^	11.95^b^	286.4^abc^
Val	11.94^c^	0.713^b^	0.676^c^	11.13^c^	306.3^a^
Ile	13.31^ab^	0.783^a^	0.697^c^	12.43^ab^	287.4^abc^
Leu	13.39^ab^	0.790^a^	0.742^a^	12.46^ab^	275.6^abc^
Trp	13.80^a^	0.815^a^	0.682^c^	13.01^a^	266.4^bc^
Arg	13.66^a^	0.799^a^	0.683^c^	12.83^a^	263.0^c^
His	13.39^ab^	0.783^a^	0.745^a^	12.39^ab^	265.6^c^
Phe + Tyr	13.65^a^	0.810^a^	0.727^ab^	12.77^a^	300.9^ab^
Gly_equi_	13.70^a^	0.802^a^	0.719^b^	12.81^a^	262.9^c^
Pro	13.20^ab^	0.794^a^	0.730^ab^	12.29^ab^	282.6^abc^
Energy	13.17^ab^	0.799^a^	0.694^c^	12.23^ab^	224.8^d^
SEM	0.281	0.0167	0.0073	0.282	12.18
*P*-value	0.002	0.010	<0.001	0.002	0.002

AME = apparent metabolizable energy; ME:GE ratio = metabolizable energy to gross energy ratio; N retention = nitrogen retention; AME_N_ = nitrogen corrected apparent metabolizable energy; Gly_equi_ = glycine equivalence.

^a-e^ Within a column, means without a common superscript were determined to be significantly different (*P* < 0.05) by a student's *t*-test.

1 Dietary treatments included 3 control diets with positive control (PC) and negative control (NC). Subsequent 9 diets are identified by their deleted amino acid 3-letter codes. Treatment means represent average of six replicate cages. The ‘Energy’ diet was created by reducing the energy content of the PC by 0.5 MJ/kg.

**Table 5:** Apparent digestibility coefficients and disappearance rates of protein in the distal jejunum and distal ileum of Ross 308 males fed diets varying in amino acid and energy supplementation 15 to 22 d in Exp. 1.

Item	Digestibility coefficients		Disappearance rates 1, g/d per bird	
	Distal jejunum	Distal ileum	Distal jejunum	Distal ileum
Treatment 2				
PC	0.784^a^	0.872^a^	19.07^abcd^	21.21^cd^
NC	0.766^ab^	0.850^abc^	20.13^ab^	22.34^bc^
Val	0.705^bcde^	0.839^bcd^	16.44^e^	19.56^e^
Ile	0.690^de^	0.840^bcd^	17.88^cde^	21.77^c^
Leu	0.694^cde^	0.843^bc^	17.84^cde^	21.68^c^
Trp	0.699^bcde^	0.814^de^	17.45^cde^	20.31^de^
Arg	0.692^cde^	0.828^cde^	17.64^cde^	21.12^cd^
His	0.740^abcd^	0.846^abc^	20.37^ab^	23.34^ab^
Phe + Tyr	0.758^abc^	0.858^ab^	19.43^abc^	21.97^c^
Gly_equi_	0.706^bcde^	0.848^abc^	18.38^bcde^	22.03^bc^
Pro	0.655^e^	0.810^e^	17.34^de^	21.39^cd^
Energy	0.718^abcde^	0.845^bc^	20.93^a^	24.63^a^
SEM	0.0240	0.0092	0.725	0.476
*P*-value	0.014	0.001	0.001	<0.001

Gly_equi_ = glycine equivalence.

^a-^^e^ Within a column, means without a common superscript were determined to be significantly different (*P* < 0.05) by a student's *t*-test.

1 Disappearance rates reflect cumulative disappearance rate for the identified intestinal section.

2 Dietary treatments included 3 control diets with positive control (PC) and negative control (NC). Subsequent 9 diets are identified by their deleted amino acid 3-letter codes. The ‘Energy’ diet was created by reducing the energy content of the PC by 0.5 MJ/kg. Treatment means represent average of 6 replicate cages.

**Table 6:** Live performance of Ross 308 males fed experimental diets from 15 to 35 d in Exp. 2.

Item	BW gain, kg	Feed intake, kg	FCR, g:g	Mortality, %
Treatment 1				
IC	2.195^bc^	2.954^bc^	1.344^abc^	0.00
PC	2.244^ab^	2.998^abc^	1.334^a^	1.39
NC	2.018^d^	2.936^c^	1.455^e^	0.00
Val	1.872^e^	2.631^d^	1.415^d^	4.17
Ile	2.138^c^	2.947^c^	1.379^c^	0.00
Leu	2.227^ab^	2.979^abc^	1.338^ab^	0.00
Trp	2.248^ab^	2.993^abc^	1.340^ab^	2.78
Arg	2.047^d^	2.939^c^	1.443^de^	1.39
His	2.223^ab^	2.960^bc^	1.343^abc^	2.78
Phe + Tyr	2.253^ab^	3.076^a^	1.374^bc^	1.39
Gly_equi_	2.259^ab^	3.027^abc^	1.351^abc^	2.78
Pro	2.254^ab^	3.076^a^	1.364^abc^	0.00
Energy	2.281^a^	3.059^ab^	1.341^ab^	0.00
SEM	0.0281	0.0379	0.0129	1.334
*P*-value	<0.001	<0.001	<0.001	0.326

BW = body weight; FCR = feed conversion ratio (feed:gain ratio); Gly_equi_ = glycine equivalence.

^a-e^ Within a column, means without a common superscript were determined to be significantly different (*P* < 0.05) by a student's *t*-test.

1 Dietary treatments included 3 control diets with industry control (IC), positive control (PC), and negative control (NC). Subsequent 9 diets are identified by their deleted amino acid 3-letter codes. Treatment means represent average of 6 replicate pens. The ‘Energy’ diet was created by reducing the energy content of the PC by 0.5 MJ/kg. Treatment means represent average of 6 replicate cages.

### 3.2 Carcass traits

Broiler chickens offered the NC diet and Arg-deleted diet had the highest relative abdominal fat pad weight; whereas birds offered the low energy diet had the lowest relative fat pad weight (*P* < 0.05) (Table 7). Broilers offered the PC diet had the highest total breast yield; whereas, broilers offered the NC diet had the lowest total breast meat yield (*P* < 0.05). The removal of supplemented Arg and Ile from the diet reduced breast yield similar (*P* > 0.05) to the NC diet, whereas, the removal of supplemented Val and Gly_equi_ resulted in total breast yield intermediate of birds fed the NC and PC diets. Birds offered all other diets did not show significant changes on total breast yield in comparison to birds fed the PC diet. Broilers fed diets devoid in Gly_equi_ had the lowest (*P* < 0.05) thigh yield; whereas all other broilers had thigh yields similar (*P* > 0.05) to birds fed the PC diet.

**Table 7:** Carcass traits of Ross 308 males fed diets varying in amino acid and energy supplementation from 15 to 35 d in Exp. 2.

Item	Parts weights			Parts yields 1		
	Total breast, kg	Thigh, kg	Fat, kg	Total breast, g/kg	Thigh, g/kg	Fat, g/kg
Treatment 2						
IC	0.621^a^	0.162^ab^	0.025^b^	226.7^abc^	59.3^bcde^	9.0^e^
PC	0.637^a^	0.163^ab^	0.028^ab^	233.6^a^	59.9^abcd^	10.3^cde^
NC	0.525^bc^	0.146^cd^	0.030^a^	211.4^e^	59.0^cde^	12.1^ab^
Val	0.514^c^	0.145^cd^	0.025^b^	219.9^cd^	61.9^a^	10.7^bcd^
Ile	0.561^b^	0.156^bc^	0.027^ab^	213.1^de^	59.1^bcde^	10.4^cde^
Leu	0.632^a^	0.164^ab^	0.027^ab^	228.5^ab^	59.5^bcde^	9.8^cde^
Trp	0.629^a^	0.163^ab^	0.026^ab^	230.7^ab^	59.9^abcde^	9.4^de^
Arg	0.531^bc^	0.143^d^	0.030^a^	216.3^de^	58.1^de^	12.2^a^
His	0.612^a^	0.162^ab^	0.030^a^	229.1^ab^	60.5^abc^	11.2^abc^
Phe + Tyr	0.620^a^	0.162^ab^	0.027^ab^	227.4^abc^	59.1^bcde^	10.0^cde^
Gly_equi_	0.618^a^	0.159^ab^	0.029^ab^	225.2^bc^	57.7^e^	10.5^cd^
Pro	0.613^a^	0.161^ab^	0.027^ab^	227.3^abc^	59.6^bcde^	10.1^cde^
Energy	0.628^a^	0.168^a^	0.018^c^	229.7^ab^	61.3^ab^	6.5^f^
SEM	0.0133	0.0039	0.0016	2.91	0.79	0.49
*P*-value	<0.001	<0.001	<0.001	<0.001	0.029	<0.001

Gly_equi_ = glycine equivalence.

^a-f^ Within a column, means without a common superscript were determined to be significantly different (*P* < 0.05) by a student's *t*-test.

1 Yields expressed relative to live body weight. Total breast yield representative of *Pectoralis major + P. minor*. Thigh representative of bone-in, skin-on right thigh.

2 Dietary treatments included 3 control diets with industry control (IC), positive control (PC), and negative control (NC). Subsequent 9 diets are identified by their deleted amino acid 3-letter codes. The ‘Energy’ diet was created by reducing the energy content of the PC by 0.5 MJ/kg. Treatment means represent average of 5 replicate cages.

### 3.3 Nutrient utilization

The effects of amino acid deletion on nutrient utilization are shown in Table 4. Only the removal of supplemented Val significantly changed AME, ME:GE ratio, and AME_N_ from those observed in broilers fed the PC diet. Broilers offered diets devoid in Energy, Arg, Ile, Trp, and Val as well as broilers fed the NC diet had significantly lower (*P* < 0.05) N retention than those fed the PC diet. Excreta dry matter was increased for broiler chickens offered diets devoid in Val and Phe + Tyr compared to those offered the PC diet.

Table 5 reports the effects of amino acid deletion on apparent protein digestibility and protein disappearance rates in the distal jejunum and distal ileum. Apparent protein digestibility in the distal ileum was significantly lower (*P* < 0.05) in broilers offered diets devoid in Energy, Arg, Ile, Pro, Trp, Leu, and Val compared to the PC diet. Broiler chickens offered Val-deleted diet had significantly lower ileal protein disappearance rate than the PC diet (*P* < 0.05); whereas broiler chickens offered His-deleted and low energy diets had significantly higher ileal protein disappearance rate than the PC diet (*P* < 0.05).

### 3.4 Plasma amino acid levels

Deletion of supplemented amino acids from the PC diet significantly influenced (*P* < 0.05) the plasma amino acid levels with the exception of Trp (*P* = 0.266) and Asp (*P* = 0.886) (Table 8, Table 9). Broilers offered Arg-deleted diet had the highest (*P* < 0.05) plasma concentration of all analyzed amino acids except Trp, Arg, Ala, Asp, and Glu. Broilers offered Leu-deleted diet had increased (*P* < 0.05) concentrations of plasma Val and Ile compared to birds fed the 3 control diets.

**Table 8:** Plasma essential amino acid concentrations (μg/mL) of Ross 308 males fed diets varying in amino acid and energy supplementation from 15 to 35 d in Exp. 2.

Item	Lys	Met	Thr	Val	Ile	Leu	Trp	Arg	Phe	His
Treatment 1										
IC	25.7^def^	13.7^e^	72.3^e^	21.0^b^	10.1^b^	17.5^bcd^	4.7	65.2^abc^	15.4^ce^	11.7^de^
PC	18.9^ef^	14.7^de^	80.3^cde^	17.4^c^	7.0^ef^	15.0^cde^	4.9	46.0^e^	16.4^bc^	7.5^fg^
NC	55.3^a^	19.6^bc^	164.1^b^	20.0^bc^	8.7^bcde^	17.3^bcd^	4.5	15.8^f^	13.3^d^	5.8^g^
Val	32.1^cd^	20.0^b^	94.5^cd^	12.1^d^	9.7^bc^	18.5^bc^	5.0	65.8^ab^	17.3^bc^	17.1^b^
Ile	24.9^def^	19.3^bc^	94.1^cd^	21.8^b^	5.4^f^	19.3^b^	5.1	67.2^a^	18.8^ab^	15.7^bc^
Leu	20.5^ef^	17.2^bcd^	88.6^cde^	32.2^a^	15.0^a^	11.5^e^	4.7	54.9^cde^	16.9^bc^	9.7^ef^
Trp	29.7^cde^	18.4^bc^	92.0^cde^	20.4^bc^	8.5^bcde^	17.1^bcd^	4.1	57.1^abcd^	17.2^bc^	11.3^de^
Arg	46.1^a^	23.6^a^	185.9^a^	32.0^a^	13.6^a^	24.6^a^	5.3	15.7^f^	20.1^a^	23.2^a^
His	26.5^cdef^	17.6^bcd^	83.7^cde^	18.8^bc^	7.9^cde^	15.8^bcd^	4.5	56.2^bcde^	15.0^cd^	5.0^g^
Phe + Tyr	17.7^f^	16.5^cde^	96.1^c^	19.8^bc^	8.4^bcde^	17.4^bcd^	4.2	53.4^de^	13.1^d^	13.3^cd^
Gly_equi_	19.1^ef^	17.6^bcd^	71.5^e^	18.8^bc^	7.5^de^	16.6^bcd^	4.5	54.8^de^	16.2^c^	10.7^def^
Pro	24.3^def^	16.8^cde^	83.7^cde^	18.3^bc^	7.3^ef^	14.7^de^	4.8	57.8^abcd^	15.4^cd^	12.1^de^
Energy	37.3^bc^	15.2^de^	73.0^de^	21.3^b^	9.4^bcd^	16.5^bcd^	5.0	65.8^ab^	15.2^cd^	13.2^cd^
SEM	4.08	1.11	7.67	1.25	0.70	1.27	0.30	3.69	0.86	1.22
*P*-value	<0.001	<0.001	<0.001	<0.001	<0.001	<0.001	0.266	<0.001	<0.001	<0.001

Gly_equi_ = glycine equivalence.

^a-^^g^ Within a column, means without a common superscript were determined to be significantly different (*P* < 0.05) by a student's *t*-test.

1 Dietary treatments included 3 control diets with industry control (IC), positive control (PC), and negative control (NC). Subsequent 9 diets are identified by their deleted amino acid 3-letter codes. The ‘Energy’ diet was created by reducing the energy content of the PC by 0.5 MJ/kg. Treatment means represent average of 2 replicate cages.

**Table 9:** Plasma conditionally essential, sparing, and non-essential amino acid concentrations (μg/mL) of Ross 308 males fed diets varying in amino acid and energy supplementation from 15 to 35 d in Exp. 2.

Item	Conditional		Sparing			Non-essential				
	Gly	Pro	Cys	Tyr	Ser	Ala	Asn	Asp	Gln	Glu
Treatment 1										
IC	46.0^cd^	49.8^bc^	15.5^bc^	28.6^fg^	46.9^d^	52.1^d^	15.5^bc^	16.4	138.7^cd^	22.7^c^
PC	44.4^d^	42.5^c^	14.3^cd^	36.9^bcde^	49.8^cd^	48.3^d^	8.7^c^	12.9	137.9^cd^	20.1^c^
NC	55.4^b^	65.9^a^	17.8^a^	24.7^gh^	61.6^b^	138.2^a^	22.5^ab^	16.7	203.0^a^	32.2^a^
Val	50.6^bcd^	54.8^b^	16.2^b^	36.4^bcde^	53.3^bcd^	52.1^d^	7.2^c^	14.8	144.0^bcd^	20.4^c^
Ile	52.4^bc^	54.1^b^	15.7^bc^	38.5^bcd^	56.3^bc^	50.2^d^	12.4^c^	15.4	149.3^bcd^	22.3^c^
Leu	47.6^cd^	54.6^b^	14.5^cd^	43.7^ab^	52.0^cd^	68.3^c^	13.5^c^	15.3	169.9^b^	23.1^c^
Trp	48.8^bcd^	48.9^bc^	15.4^bc^	40.9^abc^	51.3^cd^	53.4^cd^	7.6^c^	15.7	148.7^bcd^	22.0^c^
Arg	76.4^a^	74.9^a^	18.4^a^	47.7^a^	89.2^a^	119.4^b^	26.3^a^	14.9	217.7^a^	27.4^b^
His	48.3^bcd^	45.5^bc^	15.1^bcd^	32.5^def^	49.2^cd^	48.8^d^	10.2^c^	12.7	139.5^cd^	19.5^c^
Phe + Tyr	51.8^bcd^	53.7^b^	14.5^cd^	19.9^h^	52.1^cd^	57.0^cd^	10.8^c^	17.2	163.2^bc^	22.6^c^
Gly_equi_	34.0^e^	48.8^bc^	13.8^d^	38.2^bcd^	38.1^e^	60.5^cd^	9.2^c^	13.8	147.3^bcd^	21.2^c^
Pro	47.6^bcd^	41.5^c^	14.9^bcd^	34.9^cdef^	51.1^ce^	49.7^d^	8.4^c^	13.9	150.3^bcd^	20.8^c^
Energy	48.2^bcd^	46.5^bc^	14.6^bcd^	30.6^efg^	50.6^ce^	48.0^d^	6.9^c^	15.0	125.4^d^	20.0^c^
SEM	2.77	3.92	0.57	2.65	3.04	5.50	3.17	1.89	9.49	1.31
*P*-value	<0.001	<0.001	<0.001	<0.001	<0.001	<0.001	<0.001	0.886	<0.001	<0.001

Gly_equi_ = glycine equivalence.

^a-^^g^ Within a column, means without a common superscript were determined to be significantly different (*P* < 0.05) by a student's *t*-test.

1 Dietary treatments included 3 control diets with industry control (IC), positive control (PC), and negative control (NC). Subsequent 9 diets are identified by their deleted amino acid 3-letter codes. The ‘Energy’ diet was created by reducing the energy content of the PC by 0.5 MJ/kg. Treatment means represent average of 2 replicate cages.

## 4 Discussion

It is expected that broiler chickens offered the IC and PC diets had statistically similar growth performance including weight gain and FCR. The IC served as a benchmark representing diets currently utilized in the Australian poultry industry. Table 2 displays the calculated levels of all tested amino acids, plus Lys, Met, Cys, and Thr, a published source of requirements is also included in order to show how amino acid levels fluctuated. The cited requirements in Table 2 (Wu, 2014) were chosen as it provides requirements for 20 amino acids from a single literature source. Dietary CP were then reduced by 23.3 g/kg to gain the PC diet where the ideal protein ratios were maintained for all the relevant amino acids (Table 2). Obviously, broiler chickens offered the NC diet had significantly worse growth performance than birds offered the PC and IC diets, because the NC diet had reduced amino acid concentrations except Lys, TSAA and Thr. As variation in analyzed amino acid content was observed in the experimental diets, overall trends were in agreement with formulated levels and reductions. Apart from live performance, similar carcass traits and plasma amino acids were observed in broilers offered the IC and PC diets. In agreement with previous studies, this indicated that the supplementation of non-bound amino acids and reduction in CP from the PC diet did not influence broiler growth and protein accretion (Si et al., 2004; Chrystal et al., 2019). Furthermore, the removal of all supplemental amino acids and an energy reduction in the NC diet negatively influenced all live performance parameters and carcass traits, with the exceptions of feed intake and thigh yield. The reduction in dietary energy (0.5 MJ) did not appear to contribute the negative responses observed in broilers offered the NC diet as the energy reduction alone did not impair growth performance compared to broilers offered the PC diet.

Removal of supplemented Val had the most severe impact on live performance in both experiments. In Exp. 2, depressions in BW gain were greater in birds offered Val-deleted diet than broilers offered the NC diet. This is consistent with Sugahara et al. (1969) where deletion of a single amino acid resulted in a larger impact on performance than a simultaneous reduction in all amino acids to below requirement levels. While differences in feed conversion ratios were observed in both experiments, relative fat pad weight did not differ when compared with birds offered the PC diet. This is inconsistent with the classic relationship between FCR and relative fat pad weights, where increase of FCR often coupled with increase of abdominal fat pad weights in reduced CP diets (Chrystal et al., 2020b).

Ideal protein ratios of 20 amino acids were suggested by Wu (2014) where both ratios in the literature and whole-body protein composition were considered. The removal of supplemented Arg resulted in the largest deficiency (73%) in the diet when compared to requirement estimated by Wu (2014). Nevertheless, broilers fed diets devoid in supplemental Arg had the second lowest BW gain and impaired feed conversion in both experiments. Broiler chickens offered the Arg-deleted diet had decreased total breast meat yield coupled with increased fat pad weight compared to birds offered the PC diet. Consistently, similar responses to Arg deficiency were reported by Corzo et al. (2021) who reported the requirements of Arg to Lys ratios is 129, 116 and 109 for BW gain, feed conversion and breast yield, respectively. The deletion of Arg resulted in the largest increase in plasma Thr levels in the present study which was previously reported to occur in the face of severe amino acid deficiencies (Zimmerman and Scott, 1965) and 5 studies involved reduced CP diets (Macelline et al., 2021). Furthermore, in agreement with the findings in Zimmerman and Scott (1965), all amino acids, with the exception of Trp, accumulated in the plasma when Arg was deficient.

In Exp. 1, the removal of supplemented Ile resulted in impaired feed conversion with no impact on BW gain and feed intake. In Exp. 2, the removal of feed grade Ile reduced BW gain and feed conversion compared to broilers fed the PC diet. This inconsistency could be caused by the age differences of birds involved in both experiments. Furthermore, relative differences (compared to PC) were consistent at the conclusion of both experiments indicate that increased breast meat accretion as the bird ages (18% at 22 d vs. 24% at 35 d; Ross, 2019) despite the link between deficient Ile and decreased breast meat yield (Hale et al., 2004; Kidd et al., 2004; Tavernari et al., 2012).

Deletion of dietary His negatively influenced feed conversion during Exp. 1, but broilers in Exp. 2 showed no signs of adverse effect. Histidine is the only amino acid that can be stored in the body in the form of carnosine (Robbins et al., 1977; Hansen and Smith, 1949) and Robbins et al. (1977) suggested that the true requirement for His should be considered when muscle carnosine plateaus. In the present study, the decrease in dietary His may triggered reduction in carnosine production which may have negatively influenced feed conversion until such time that birds adjusted to the lower His diet.

The removal of Trp resulted in decreased BW gain, increased feed intake and feed conversion ratio in Exp. 1, but in Exp. 2 no adverse effects were observed for broilers fed Trp-deleted diet. Existing literature on the Trp requirement indicates that a Trp to Lys ratio of 19 is necessary to obtain optimal performance, especially for the first 3 weeks (Cozo et al., 2005; Corzo, 2012); whereas a requirement could not be determined for optimal performance during a 22 to 42 d growing period (Duarte et al., 2013). This suggested a decreasing requirement of His with age and the removal of feed grade Trp in the present study only led to 3% deficiency. Interestingly, very little variation in plasma Trp levels among different dietary treatments were observed. Therefore, any deficiency that could have occurred early on in the 15 to 22 d period, may have been overcome as the growout period continued to 35 d post–hatch.

Influence of the removal of supplemented Leu was limited to a marginal increase in feed intake (37 g) and a numeric increase in BW gain (36 g) compared to birds fed the PC in Exp. 1. Although decreases in protein digestibility were observed in both distal jejunum and distal ileum, the marginal increase in feed intake allowed for comparable protein disappearance rates. Furthermore, these trends were also observed in Exp. 2 with no influence on performance or carcass traits. Interestingly, despite the lack of negative effects on performance parameters, free Val and Ile accumulated in the plasma when Leu was deficient. The branched-chain amino acids share a common degradation pathway for the first two steps of their catabolism, in which they undergo a reversible transamination followed by an irreversible decarboxylation (Harper et al., 1984). These shared steps have been identified as a potential mode of action for the antagonism observed among the branched-chain amino acids where a dietary excess of any of the 3 branched-chain amino acid can lead to the inadvertent catabolism of the other 2 branched-chain amino acids (Maynard et al., 2021a). The buildup of plasma Val and Ile in a diet deficient in Leu may indicate that dietary Leu may play a larger role in branched-chain amino acid catabolism than Val and Ile. Previous data on amino acid catabolism indicated that dietary protein level largely controls the amino acid catabolism enzyme activity as opposed to the levels of individual amino acids *per se* (Keene and Austic, 2001). In typical poultry diet, Leu is the most abundant branched-chain amino acid and given the possibility that Leu plays bigger role in branched-chain amino acid catabolism, Leu excess may be the most potent cause of branched-chain amino acid antagonism (Mathieu and Scott, 1968; D'Mello and Lewis, 1970; Harper et al., 1984).

The removal of Phe + Tyr had no negative impact on performance or carcass traits except a minor increase in feed conversion ratio (e.g., 4 points increase compared with PC). While the majority of Phe and Tyr requirement studies are dated and focus on young chicks (Sasse and Baker, 1972; Baker and Han, 1994; Sterling, 2000; Lartey and Austic, 2008; Franco et al., 2017), Lartey and Austic (2008) found that the Phe requirement was lower for BW gain than feed conversion ratio. In the present study, the removal of Phe + Tyr did not lead to deficiency in these amino acids due to the higher level of Phe + Tyr derived from intact protein ingredients; hence, significant impact on growth performance was not observed.

Both Pro and Gly are conditionally limited especially during times of rapid growth when the chick cannot produce enough *de novo* to supply their respective requirements. The requirement of Gly coupled with Ser, which often met by Gly supplementation, is either expressed as Gly + Ser inclusions (Dean et al., 2006) or Gly equivalent (Siegert et al., 2015a). The inclusion of excess Ser in the diet has been shown to completely eliminate the need for Gly in the diet (Baker et al., 1968), and due to the efficient molar conversion of Ser to Gly, the dietary levels of Gly + Ser can be expressed as Gly_equi_ to more accurately represent the amount of molar Gly in the diet (Siegert et al., 2015b). Glycine requirement studies typically utilized young birds and determined the requirement when the most rapid growth occurs; therefore, the production of Gly from other nutrients, such as Thr and choline may not be efficient to satisfy Gly requirements (Coon et al., 1974; Maruyama et al., 1978; Waldroup et al., 2005a; Siegert et al., 2015a). In the present study, Gly_equi_ showed no signs of limiting growth other than a minor increase in feed intake in Exp. 1; whereas, in Exp. 2, its deletion resulted in reduced breast and thigh yield compared to birds fed the PC diet.

The reduction of dietary energy by 0.5 MJ/kg had no negative effect on growth performance and carcass yield but reduced relative fat pad weights. Similarly, recent studies reported energy reduction ranged from 0.29 to 0.42 MJ/kg had minimal impact on feed intake and no effect on carcass traits (Gopinger et al., 2017; Maynard et al., 2019). Although distal ileal protein digestibility coefficients were reduced in birds offered diets with reduced energy content, protein disappearance rates in the distal ileum were increased relative to the PC diet. Despite the reduced protein digestibility, the reduction in dietary energy led to a 12.6 g/kg decrease in soybean oil inclusion. Soybean oil represents an energy dense feed ingredient that is commonly used to satisfy energy values in formulation but is also a high-cost ingredient (Barbour et al., 2006). The result indicates that feed cost may be reduced by decreasing added oil inclusion without compromising live performance in low-CP diet.

## 5 Conclusions

Overall, Val and Arg are likely co-limiting in wheat-soybean meal diets followed by Gly_equi_ and Ile. Limitations in branched chain amino acids, especially Val and Ile compromised weight gain, FCR and breast meat yield to the largest extent when compared with limitation of other amino acids in reduced CP diets based on wheat. Gly and Ser limitation significantly compromised FCR whereas limitation in His significantly reduced breast meat and thigh meat yield in reduced CP diets without influencing FCR and weight gain. It is encouraging that reducing energy level by 0.5 MJ/kg in the 180 g/kg CP diet reduced fat pad weights without compromising growth performance. Further investigations are required to confirm this finding as reduction in dietary energy has strong implication on reducing feed cost.

## Author contributions

**Craig W. Maynard** contributed to feeding study supervision, data collection, data analyses and the original manuscript. **Michael T. Kidd** contributed to experimental design. **Peter V. Chrystal** contributed to experimental design and diet formulation, **Leon R. McQuade** and **Bernie V. McInerney** contributed to amino acid analyses. **Peter H. Selle** contributed to experimental design, **Sonia Y. Liu** contributed to funding acquisition, experimental design and data analyses. All authors contributed to reviewing, writing and editing of the manuscript.

## Declaration of competing interest

We declare that we have no financial and personal relationships with other people or organizations that can inappropriately influence our work, and there is no professional or other personal interest of any nature or kind in any product, service and/or company that could be construed as influencing the content of this paper.
